# Hydrogen selenide in biological systems: from synthetic donors to fluorescent probes

**DOI:** 10.1039/d6sc05851e

**Published:** 2026-08-27

**Authors:** Yibo Zhou, Pin Shang, Jun Zhang, Zhihe Qing, Tony D. James

**Affiliations:** a Hunan Provincial Key Laboratory of Cytochemistry, School of Chemistry and Pharmaceutical Engineering, Changsha University of Science and Technology Changsha 410114 P. R. China yibozhou@163.com qingzhihe@hnu.edu.cn; b Department of Chemistry, University of Bath Bath BA2 7AY UK; c School of Chemistry and Chemical Engineering, Henan Normal University Xinxiang 453007 China t.d.james@bath.ac.uk

## Abstract

Analogous to hydrogen sulfide (H_2_S), the more strongly reductive hydrogen selenide (H_2_Se) functions as a key endogenous mediator, exerting significant influence on signal transduction, redox regulation, and cytoprotection. However, the *in vivo* generation of H_2_Se *via* molecular donors and the real-time monitoring of its fluctuations remain challenging due to inherent instability. To tackle these challenges, several H_2_Se donors, including organic small molecule, inorganic salt, and composite nanoparticles, have been developed to ensure controlled H_2_Se delivery. Meanwhile, various kinds of fluorescent probes have been developed as powerful tools for the real-time visualization of H_2_Se at both cellular and organismal levels. This review will summarize the development of H_2_Se donors and fluorescent probes and emphasize the underlying obstacles that still exist in this field, with the aim of providing a deeper understanding of the biological functions of endogenous H_2_Se and facilitate its exploration across interdisciplinary fields.

## Abstract 2

In this review, the recent developments in H_2_Se donors and fluorescent probes are reviewed from the perspectives of molecular design, response mechanisms, and biological applications, followed by an overview of the challenges in H_2_Se research.

## Graphical abstract

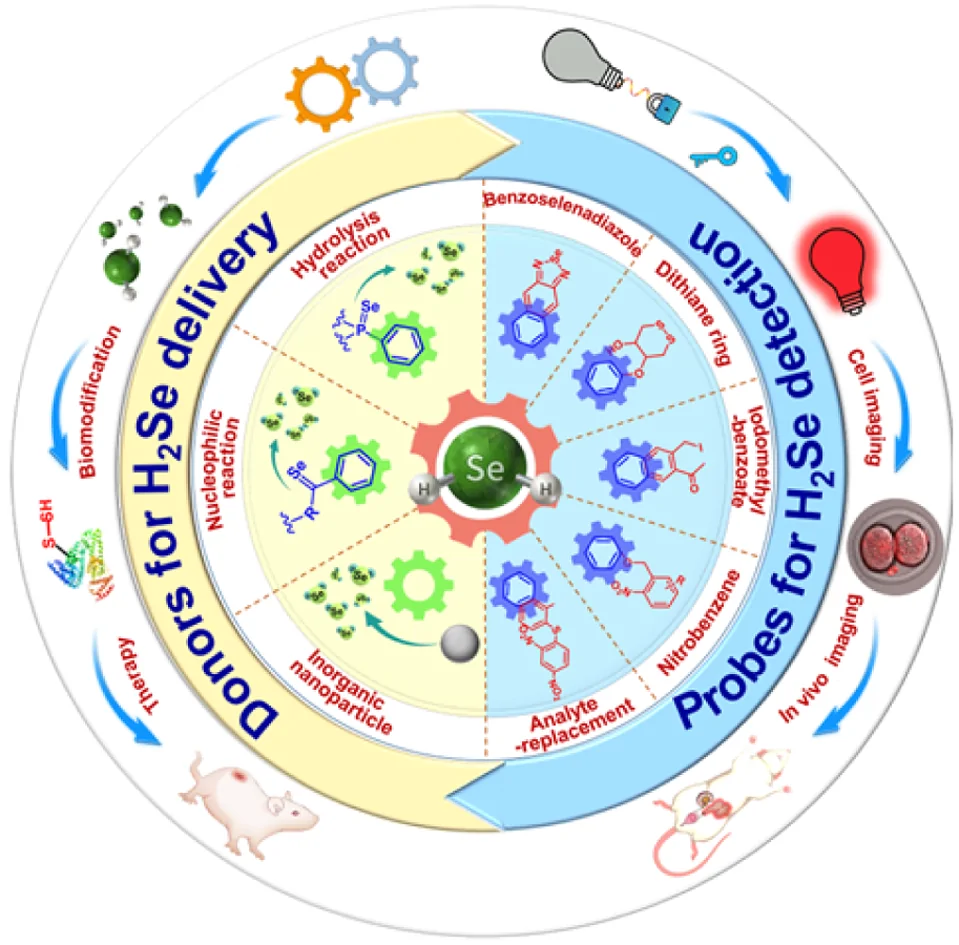

## 1. Introduction

As a vital member of the chalcogen group, selenium (Se) was firstly identified in mammals in the 1950s,^1^ establishing its status as a fundamental element of life. Se deficiency is associated with a range of disorders, such as Keshan disease and Kashin–Beck disease.^2,3^ While excessive Se intake is closely linked to neurological disorders,^4,5^ skin disease,^6^ and dental diseases.^7^ Thus, the dose-dependent paradox highlights the double-edged sword of Se in physiological processes.^8^ It has been reported that numerous proteins *in vivo* contain selenocysteine (SeCys) residues,^9^ which include redox-regulating thioredoxins,^10,11^ selenium-transporting proteins,^12^ and iodothyronine deiodinases essential for thyroid metabolism.^13^ The functional diversity and essential nature of these selenoproteins provide robust evidence for the indispensable role of Se in maintaining physiological homeostasis.^14^

Dietary intake is the primary route for Se supplementation in humans, through which Se is primarily ingested as selenomethionine (SeMet) from botanical sources and SeCys from animal tissues.^15–17^ The environmental availability of Se in soil exhibits marked spatial heterogeneity, leading to substantial global disparities in its human intake. For instance, a prominent Se-deficient belt stretches diagonally from Northeast to Southwest China.^18^ In these areas, Se concentrations across the ‘soil-crop-human’ axis typically fall below 20 µg kg^−1^,^19^ which fails to meet the WHO-recommended threshold of 50–100 µg kg^−1^.^20^ Chronic inadequacy of Se intake diminishes the catalytic activity of essential selenoproteins, such as glutathione peroxidase, which ultimately impairs the cellular antioxidant network.^21,22^ Thus, developing new cultivars of selenium-fortified crops represents a critical strategy for the supplementation of selenium and the maintenance of human health.^23,24^

Hydrogen selenide (H_2_Se) serves as a key metabolic intermediate of Se metabolism *in vivo*, which can be further converted into SeCys, selenouridine, methylselenide, and other Se-containing biomolecules.^25–27^ In terms of molecular geometry, H_2_Se is structurally analogous to hydrogen sulfide (H_2_S), a well-established and extensively studied signalling molecule belonging to the same chalcogen group.^28,29^ In particular, Se atom possess increased size and polarizability compared with the S atom, thereby H_2_Se exhibits enhanced reactivity toward intracellular proteins,^30,31^ exerting a potent influence on various signalling pathways even at extremely low concentrations. Accordingly, H_2_Se has been recognized as the fourth gasotransmitter following NO, CO, and H_2_S,^32–35^ attracting extensive research interest in biological and chemical fields.

## 2. Physiological functions of H_2_Se

In biological systems, H_2_Se can be generated through various metabolic pathways. For instance, SeMet can substitute for methionine during protein synthesis and subsequently be converted into SeCys *via* the transsulfuration pathway,^36^ and the SeCys is then further converted into H_2_Se by selenocysteine β-lyase.^37^ Inorganic selenites can also undergo reductive metabolism to generate H_2_Se.^38,39^ Specifically, exogenous selenite initially undergoes a non-enzymatic reaction with GSH to form selenodiglutathione (GS-Se-SG), which subsequently experiences stepwise reduction catalyzed by glutathione reductase, ultimately generating H_2_Se as a bioavailable selenium pool. Given the strict dependence of each metabolic step on a sustained GSH supply, the intracellular GSH level acts as the direct driver of H_2_Se generation efficiency and homeostasis.^40,41^

H_2_S mediates the oxidative modification of protein thiols to form persulfide groups (–SSH), a post-translational modification that directly affects protein activity and governs key signalling pathways in various physiological contexts.^42^ As such research on H_2_Se has similarly been pursued. H_2_Se facilitates the covalent selenylation of pivotal redox-sensitive enzymes, including thioredoxin and glutathione peroxidase (GPx), which in turn modulates intracellular redox homeostasis.^43–45^ Additionally, there is a close relationship between H_2_Se status and the incidence of diseases. For instance, in transgenic mouse models of Alzheimer's disease (AD), a deficit in endogenous H_2_Se has been found to trigger pronounced amyloid plaques deposition, thereby aggravating the progression of the disease.^46,47^

Meanwhile, H_2_Se can rapidly react with excessive reactive oxygen species (ROS) to mitigate oxidative stress,^48,49^ and it can bind with toxic heavy metals (Hg^2+^) for treatment of arsenic poisoning.^50,51^ In addition, its ability to interact with heme Fe^2+^ facilitates the transient inhibition of cytochrome c oxidase activity,^52,53^ further highlighting its complex physiological influence. Concurrently, the depletion of GSH represents a widespread paradigm to sensitize malignant cells to oxidative stress-induced apoptosis.^54,55^ Given that the biosynthesis of H_2_Se inherently consumes endogenous GSH, the intracellular H_2_Se level serves as an indirect indicator of the cellular physiological state. Furthermore, it can also reflect the therapeutic efficacy of co-administered GSH-depleting therapeutic agents. Therefore, various kinds of H_2_Se donors have been developed to deliver H_2_Se into diseased regions for therapy.^56^ Accordingly, fluorescent probes have been developed to image endogenous H_2_Se dynamics in real time to evaluate pathological changes.

**Fig. 1:**
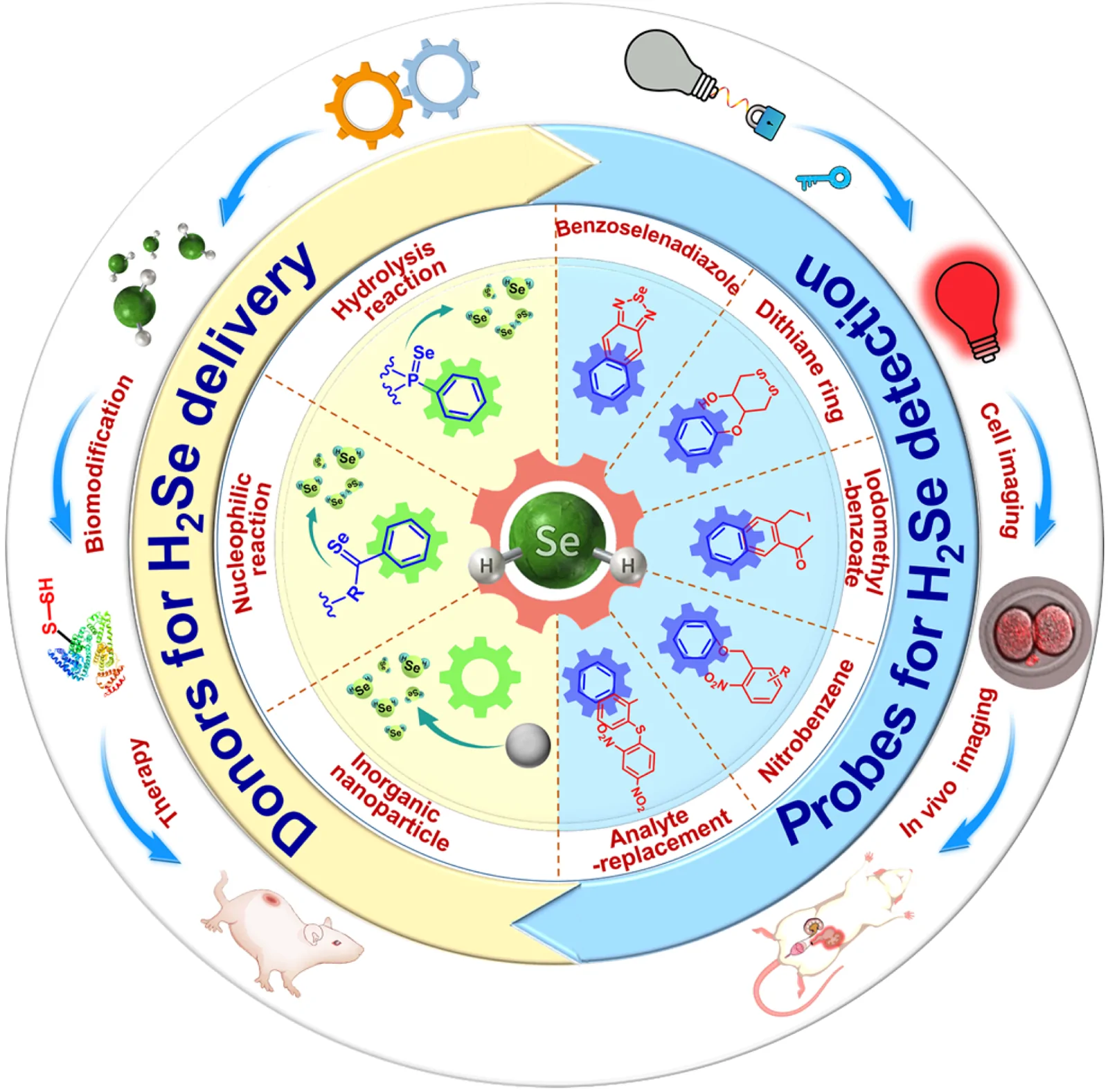
Schematic overview of H_2_Se donors and fluorescent probes and their application in the delivery and detection of H_2_Se in biological system.

This review is structured into two sections: H_2_Se donors and fluorescent probes (Fig. 1). First, molecular donors based on hydrolytic and nucleophilic reactions, and inorganic nanoparticles have been developed for H_2_Se delivery *in vivo*. Therefore, we will elaborate on the molecular design, activation mechanisms, and transport strategies of these donors, and summarize the intracellular signalling pathways of H_2_Se in functional recovery. Then, we will focus on the design and application of H_2_Se-activated probes, including the design principles and response mechanisms of fluorescent probes will be systematically elaborated, and the intracellular distribution and real-time fluctuations of H_2_Se will be elucidated. Taken together, this review provides a comprehensive overview of current H_2_Se research, providing information on the intricate biological functions and establishing a robust framework for the future development of H_2_Se delivery and detection.

## 3. Synthesis and biological application of H_2_Se donors

By virtue of the essential role of H_2_Se in human physiology, a consistent and adequate intake of Se is essential to provide the necessary precursors for H_2_Se biosynthesis. For instance, the Se content in certain nuts can reach as high as 25–76 mg kg^−1^,^57^ and there is also a high concentration of Se in mushrooms (0.085–48.5 mg kg^−1^).^58,59^ Thereby foods that contain Se have garnered increasing attention in recent years.

Apart from Se-enriched foods, developing efficient exogenous donors capable of directly delivering H_2_Se has emerged as a promising new strategy for satisfying the need of the human body. Recently, various H_2_Se donors based on different reaction mechanisms have been developed for the generation of H_2_Se *in vivo*. In the following section, we will provide a comprehensive summary and analysis of currently reported H_2_Se donors.

### 3.1 H_2_Se donors based on hydrolysis reactions

**Fig. 2:**
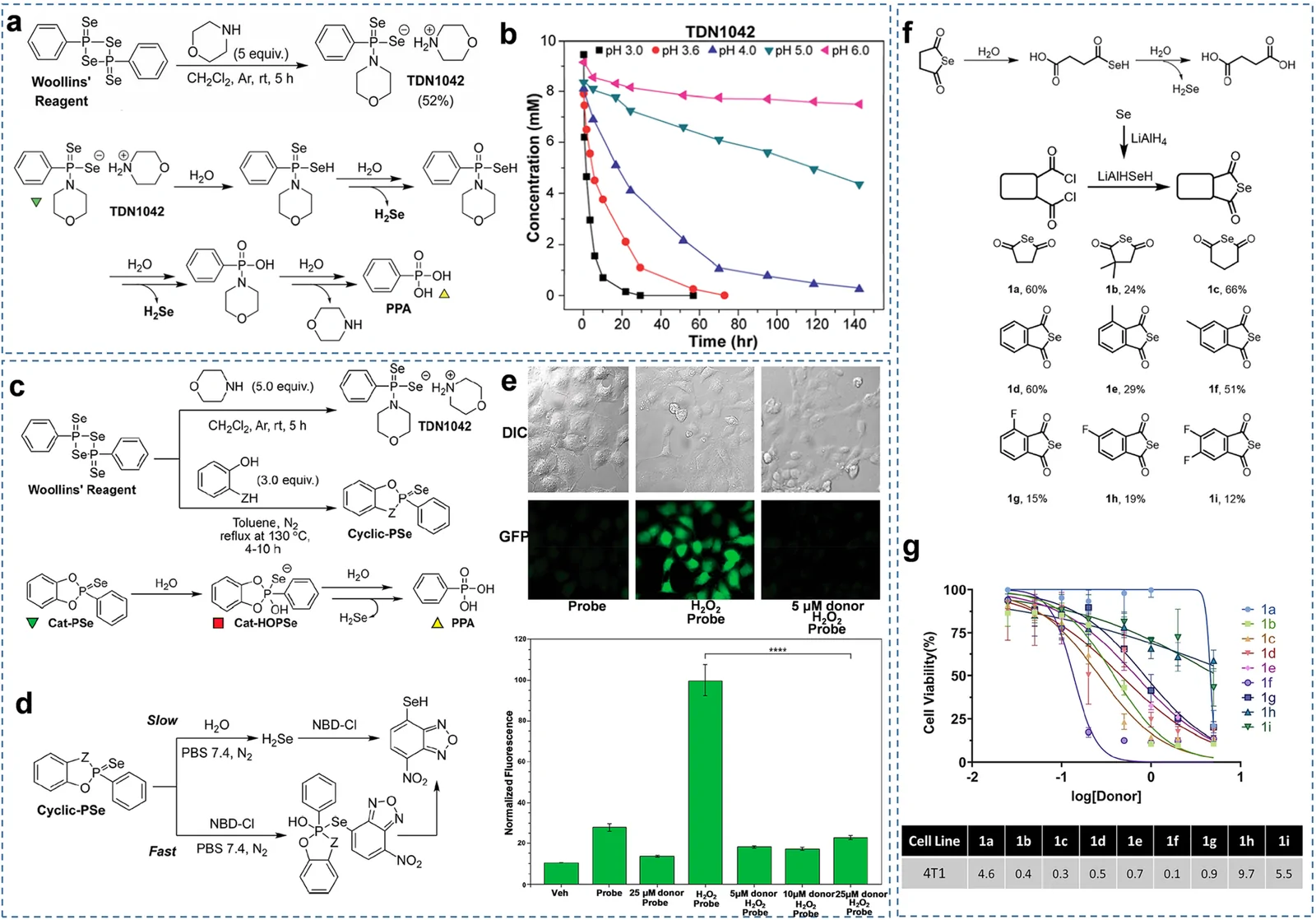
(a) Synthetic route and hydrolysis mechanism of chemical donor (TDN1042). Reproduced with permission from ref. 61. Copyright 2019, Royal Society of Chemistry. (b) The reaction kinetics of TDN1042 in different pH solutions. Reproduced with permission from ref. 61. Copyright 2019, Royal Society of Chemistry. (c) Synthetic route and hydrolysis mechanism of H_2_Se donor based on cyclized P=Se bond. Reproduced with permission from ref. 62. Copyright 2021, American Chemical Society. (d) Reaction mechanism of Cyclic-PSe in H_2_O and NBD-Cl. Reproduced with permission from ref. 62. Copyright 2021, American Chemical Society. (e) Fluorescence images of cells treated with H_2_O_2_ and H_2_Se donor. Reproduced with permission from ref. 62. Copyright 2021, American Chemical Society. (f) Hydrolysis mechanism of chemical donor based on phthalic selenoanhydride, and the molecular structure of different donors. Reproduced with permission from ref. 63. Copyright 2025, Royal Society of Chemistry. (g) Survival rate of the live cells treated with different kinds of H_2_Se donors. Reproduced with permission from ref. 63. Copyright 2025, Royal Society of Chemistry.

It has been reported that H_2_S could be effectively released through the hydrolysis of P=S bonds.^60^ Inspired by this, Pluth's group synthesized a chemical donor (TDN1042) through the reaction of Woollins' reagent with morpholine (Fig. 2a, top),^61^ which generates H_2_Se by the hydrolysis reaction of the P=Se bond. The NMR results indicated that TDN1042 was converted into phenylphosphonic acid (PPA) during the hydrolysis process, confirming the hydrolytic cleavage of the P=Se bond (Fig. 2a, bottom). Furthermore, the hydrolysis rate increased as the pH decreased (Fig. 2b). Considering the acidic conditions present in tumor regions, this pH-dependent characteristic indicates that TDN1042 is suitable for the treatment of cancer due to an enhanced ability for H_2_Se release.

Although the aforementioned donors enable a sustained and slow release of H_2_Se *via* hydrolysis reactions, the limited hydrolysis rates hinder practical applications. To address this issue, Pluth's group optimized the performance of H_2_Se donors based on P=Se bonds.^62^ As shown in Fig. 2c (top), compared with the previous TDN1042, *ortho*-substituted phenols were introduced to construct a cyclized P=Se donor (Cyclic-PSe). With addition of H_2_O to Cyclic-PSe, the phosphorus center is nucleophilically attacked by H_2_O. Then H_2_Se is rapidly released by protonation of the anionic selenide (Fig. 2c, bottom). To confirm the hydrolysis mechanism of Cyclic-PSe, 4-chloro-7-nitrobenzofurazan (NBD-Cl) was added to react with Cyclic-PSe to generate a fluorescence signal. As shown in Fig. 2d, NBD-Cl can directly attack the P=Se bond to form the chromogenic final product. Compared with the hydrolysis reaction, the electrophilic nature of NBD-Cl guaranteed a high reaction rate. To demonstrate the antioxidant properties of this donor, intracellular imaging experiments were performed. With the ROS probe as indicator, the incubation of Cyclic-PSe with cells pretreated with hydrogen peroxide (H_2_O_2_) can markedly reduce the fluorescence signal intensity (Fig. 2e), further confirming its capacity for intracellular ROS scavenging.

The structural complexity and tedious synthetic routes towards H_2_Se donors further restricts their large-scale production and use. Pan's group reported a facile synthesis of H_2_Se donors based on phthalic selenoanhydride.^63^ As illustrated in Fig. 2f (top), the cyclic selenoimide first undergoes hydrolytic ring-opening to generate a succinyl monoselenol intermediate containing a selenol group (–SeH). Subsequent cleavage of the C–Se bond leads to the release of H_2_Se and the formation of succinic acid. Meanwhile, to elucidate the effect of substituent groups on the hydrolysis rate, a series of structurally related H_2_Se donors bearing different substituents were designed and synthesized (Fig. 2f, bottom). The results indicated that the hydrolysis rate is governed by molecular structure: aliphatic ring strain effectively accelerates the ring-opening process, while aromatic substituents modulate activity through electronic effects. Specifically, electron-donating groups stabilize the selenanhydride structure, thereby reducing the reaction rate, whereas electron-withdrawing groups markedly enhance hydrolytic activity. To further confirm the practical application of these donors, live cells (4T1 cells) were treated with these chemical tools. Cell viability assays confirmed that these selenoimide-based H_2_Se donors exhibited varying degrees of growth inhibition in cells (Fig. 2g), suggesting the potential for applications in anti-tumor therapy.

Hydrolysis-triggered H_2_Se donors represent a foundational and widely explored class, celebrated for their capacity to continuously generate H_2_Se under physiological conditions without requiring exogenous stimuli. However, pioneering P=Se-based donors suffer from slow hydrolysis rates, which may limit their biological efficacy. Although structural optimizations, such as cyclization or electronic modulation, can significantly accelerate H_2_Se release, most hydrolysis-responsive donors still lack precise spatiotemporal control, making them susceptible to premature activation prior to reaching target sites. Therefore, balancing release efficiency, structural simplicity, and biological specificity remains a key consideration in the design of hydrolysis-based H_2_Se donors.

### 3.2 H_2_Se donors based on nucleophilic reactions

In addition to the hydrolysis mechanism, triggering of H_2_Se release *via* nucleophilic substitution or addition reactions has emerged as another vital chemical pathway. Given the differential distribution of endogenous species in physiological and pathological processes, the design and synthesis of artificial small-molecule donors that can be specifically activated by endogenous triggers, such as biothiols and enzymes,^64^ have emerged as a core research focus in the field of H_2_Se chemical biology. By virtue of precise molecular design, these donors enable the site-specific and real-time release of H_2_Se within complex physiological environments, thereby providing robust molecular toolkits to elucidate the physiological and pathological roles of H_2_Se.

**Fig. 3:**
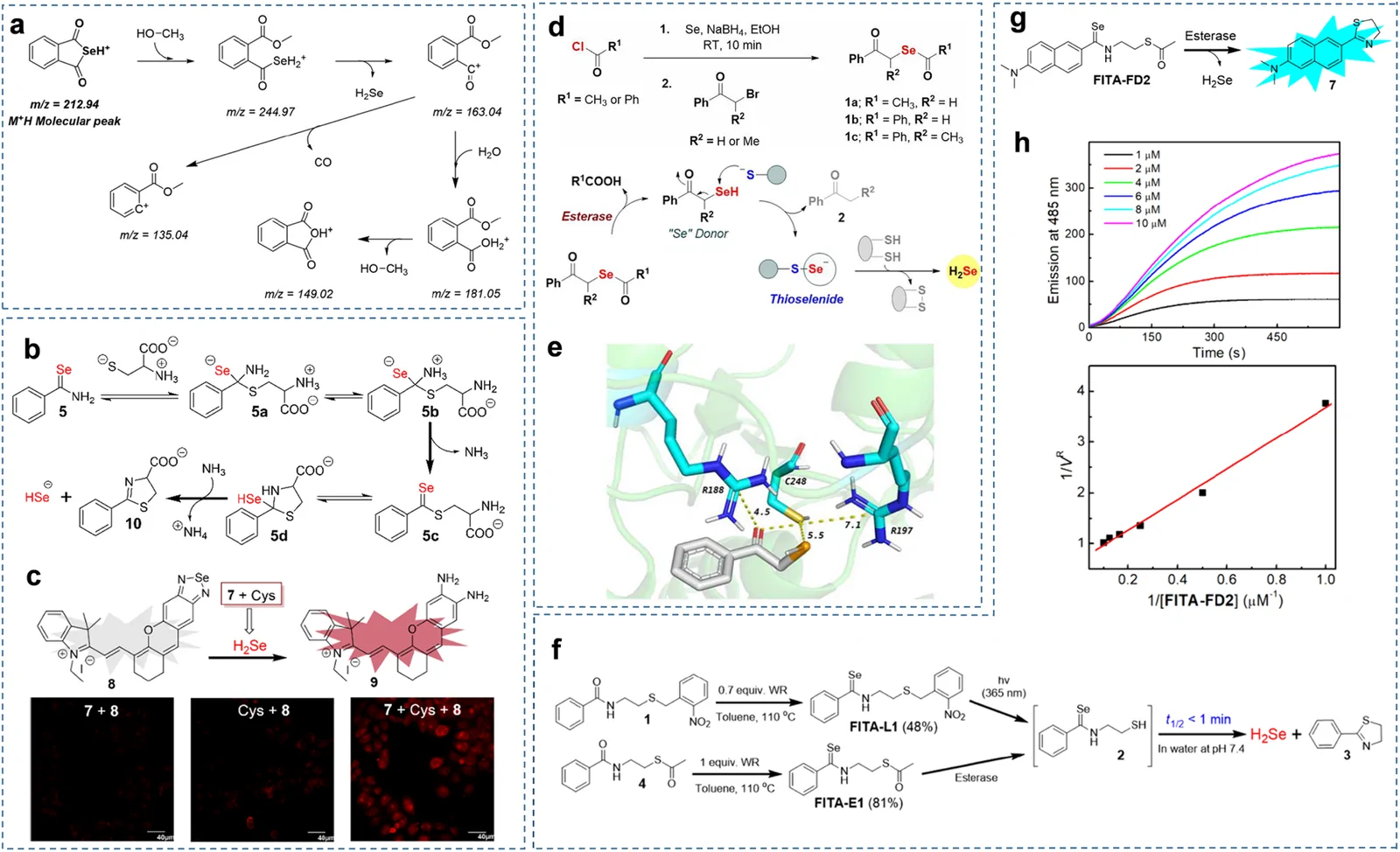
(a) Reaction mechanism of H_2_Se donor based on phthalic selenanhydride. Reproduced with permission from ref. 65. Copyright 2019, Royal Society of Chemistry. (b) The reaction mechanism of H_2_Se donor based on selenoamide response to Cys. Reproduced with permission from ref. 67. Copyright 2022, American Chemical Society. (c) Fluorescence images of cells treated with fluorescent probe under different conditions: donor, Cys, donor + Cys. Reproduced with permission from ref. 67. Copyright 2022, American Chemical Society. (d) The synthesis of H_2_Se donors based on phenacylselenoesters from acyl chloride derivatives (top), and the reaction mechanism of H_2_Se donor response to esterase (bottom). Reproduced with permission from ref. 68. Copyright 2024, Royal Society of Chemistry. (e) Molecular docking analysis of the active site in protein and H_2_Se donor. Reproduced with permission from ref. 68. Copyright 2024, Royal Society of Chemistry. (f) Synthesis of stimuli-responsive donors and their mechanistic pathways for H_2_Se release. Reproduced with permission from ref. 69. Copyright 2024, American Chemical Society. (g) Reaction mechanism of fluorophore-containing H_2_Se donor towards esterase. Reproduced with permission from ref. 69. Copyright 2024, American Chemical Society. (h) Time-dependent fluorescence changes of H_2_Se donor after being treated with different concentrations of esterase, and the corresponding Lineweaver–Burk plots to obtain Michaelis constant (*K*_m_). Reproduced with permission from ref. 69. Copyright 2024, American Chemical Society.

In 2019, Domínguez-Álvarez and co-workers reported phthalic selenanhydride as a highly efficient H_2_Se donor.^65^ As illustrated in Fig. 3a, the acyl-selenium (C–Se) bonds within phthalic selenanhydride exhibit relatively low stability, which renders them highly susceptible to nucleophilic attack by endogenous species, such as H_2_S (in the form of HS^−^ or S^2−^) and GSH. Initiated by a site-specific nucleophilic attack on the carbonyl carbon or selenium atom, the selenanhydride undergoes a substitution-driven cleavage of the C–Se bond, leading to the subsequent release of Se^2−^. Under physiological conditions, the Se^2−^ undergoes rapid protonation and is ultimately converted into H_2_Se. This release mechanism has been validated by mass spectrometric (MS) analysis and redox experiments. Furthermore, these selenanhydrides exhibit exceptional selectivity and anti-proliferative activity against live cancer cells, highlighting their promise as lead compounds for the development of novel anti-tumor agents.

Specific residues within the protease active site can also function as nucleophiles. For instance, Krakowiak's group developed a novel H_2_Se donor based on 2′-deoxyguanosine-5′-*O*-selenophosphate (dGMPSe),^66^ which undergoes nucleotide-binding protein 1 (HINT1)-mediated enzymatic hydrolysis to release H_2_Se *in situ*. The reaction mechanism of dGMPSe indicates that the imidazole nitrogen of the histidine residue in HINT1 initiates a nucleophilic attack on the phosphorus atom of the substrate dGMPSe, forming a phosphorylated enzyme intermediate (HINT1-dGMP). Then the P–Se bond undergoes cleavage to release selenide (Se^2−^), a step identified as the rate-determining step of this nucleophilic reaction. Finally, Se^2−^ abstracts protons from H_2_O_2_ or HPO_4_^2−^ to generate H_2_Se. The development of dGMPSe provides a novel strategy for generating the H_2_Se *in vivo*.

Also, biothiols can act as nucleophiles to induce the generation of H_2_Se. Yi's group designed and synthesized novel H_2_Se donors based on selenocyclopropenone and selenoamide scaffolds.^67^ As shown in Fig. 3b, the sulfhydryl group of Cys initiates a nucleophilic attack on the C=Se bond to form a tetrahedral intermediate, which further undergoes electronic rearrangement to yield intermediate 5b. Following the spontaneous release of one equivalent of NH_3_, 5b is converted into 5c. Then an intramolecular nucleophilic reaction occurs to form the tetrahydrothiazole derivative 5d. Due to its instability, 5d undergoes the elimination of HSe^−^ to produce the stable dihydrothiazole 10. The feasibility of this donor was further confirmed in live cells. As shown in Fig. 3c, with a H_2_Se fluorescent probe as indicator, a significant signal was observed only in cells pre-treated with both the donor and Cys, thereby demonstrating that this donor can be effectively applied for the intracellular release of H_2_Se.

Chakrapani's group reported a new strategy to generate H_2_Se based on the enzymatic reaction of phenacylselenoesters.^68^ As shown in Fig. 3d (top), selenoester precursors (1a–1c) can be synthesized *via* acyl chloride reactions with α-haloketones. In the presence of esterases, the benzoyl selenoester is cleaved by esterase to generate phenacylselenol, which subsequently undergoes nucleophilic attack by thiols to form thioselenide species. These thioselenide intermediates are further reduced to produce H_2_Se or react with cysteine residues on proteins to form protein thioselenides (Fig. 3d, bottom). Molecular docking analysis proves that the selenium centre, much like its sulfur counterpart, possesses exceptional reactivity with the catalytic cysteine (Fig. 3e), further proving the pivotal role of H_2_Se within complex biological environments. Simultaneously achieving the release and monitoring of H_2_Se is of great significance. To achieve this goal, Yi's group constructed a general H_2_Se donor platform based on intramolecular aryl thiol activation (FITA).^69^ This system leverages intramolecular thiol activation of aryl selenoamides to achieve rapid and efficient release of H_2_Se, with a half-life (*t*_1/2_) of less than 1 min. As illustrated in Fig. 3f, the donor undergoes *in situ* generation of a thiolate anion intermediate upon photo-irradiation or enzymatic reaction. This intermediate subsequently performs a rapid nucleophilic attack on the intramolecular selenoamide site, forming a tetrahedral intermediate. Following protonation and further dissociation, H_2_Se is ultimately released. Furthermore, by changing the scaffold to a coumarin, the first fluorescence-containing H_2_Se donor was successfully constructed (Fig. 3g). Different from the previous H_2_Se donor, the generation of H_2_Se was accompanied by the generation of a fluorescence signal, enabling more intuitive monitoring of the H_2_Se generation kinetics. From the time-based emission result, the fluorescence intensity increased with the esterase concentration (Fig. 3h), and the kinetic constant was in good agreement with those of the substrates, which further demonstrates the H_2_Se-generation capacity of this donor. This strategy provides a general platform for activation by different targets using different recognition groups, and it also enables real-time visualization and monitoring of H_2_Se release kinetics.

**Fig. 4:**
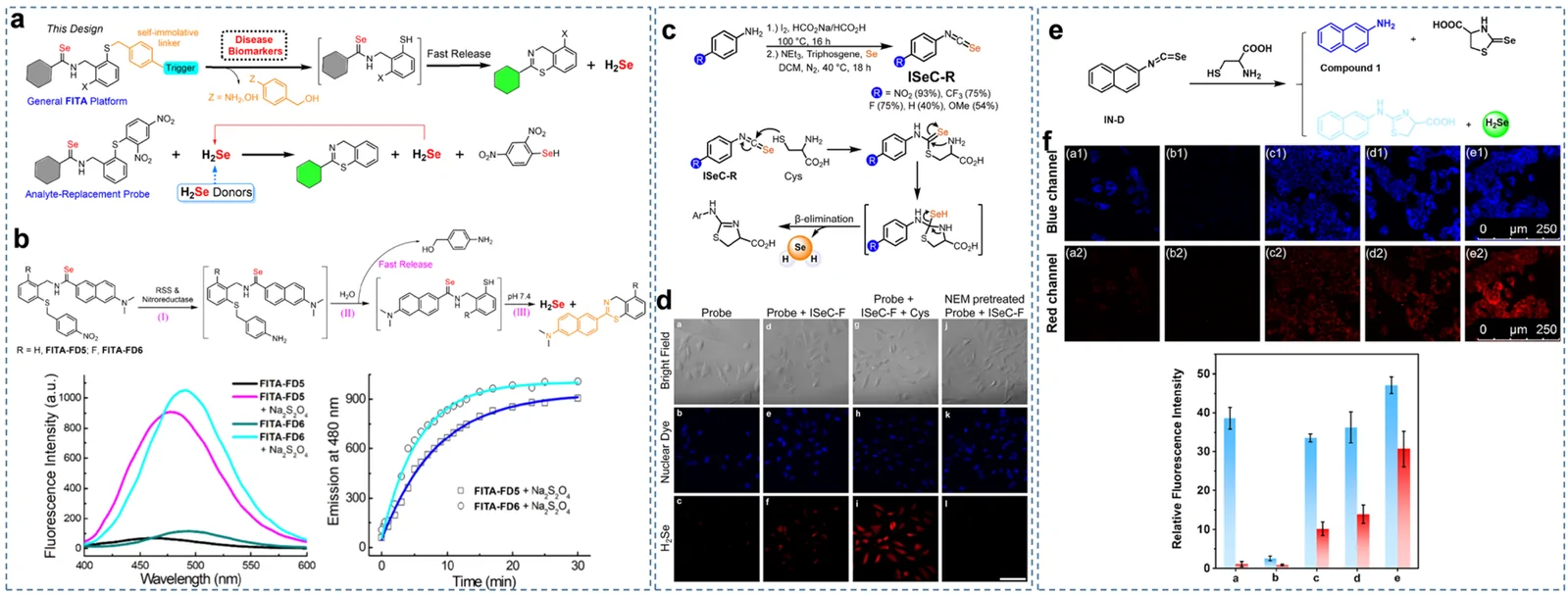
(a) Reaction mechanism of H_2_Se donor based on analyte-replacement strategy. Reproduced with permission from ref. 70. Copyright 2025, American Chemical Society. (b) Bifunctional donor-probe molecular tool for liberating H_2_Se (top), and the fluorescence emission spectrum of donors with and without sodium selenite. Reproduced with permission from ref. 70. Copyright 2025, American Chemical Society. (c) The synthesis and reaction mechanism of H_2_Se donor based on isoselenocyanates. Reproduced with permission from ref. 71. Copyright 2025, Royal Society of Chemistry. (d) Fluorescence images of cells treated with fluorescent probe under different conditions: donor, Cys + donor, NEM + Cys + donor. Reproduced with permission from ref. 71. Copyright 2025, Royal Society of Chemistry. (e) Reaction mechanism of isoselenocyanate-based donor to Cys. Reproduced with permission from ref. 72. Copyright 2025, Elsevier. (f) Fluorescence images of cells treated with IN-D and fluorescent probe under different conditions. The blue and red fluorescence represent the IN-D and H_2_Se fluorescent probe, respectively. Reproduced with permission from ref. 72. Copyright 2025, Elsevier.

While the aforementioned research enables the simultaneous release and detection of H_2_Se, the real-time detection mechanism inherently leads to the consumption of the produced H_2_Se. To further address this challenge, Yi's group developed novel H_2_Se donors based on an analyte-replacement strategy.^70^ As shown in Fig. 4a (top), upon specific activation of the recognition site by disease biomarkers, a cascade cleavage reaction is triggered to release an aryl thiol. The liberated thiol then undergoes an intramolecular nucleophilic cyclization with the adjacent aryl selenoamide, yielding a selenated cyclic product and the synchronous release of H_2_Se. This design offers the distinct benefit of monitoring H_2_Se without depleting the original pool of the analyte (Fig. 4a, bottom), thereby ensuring the integrity of cellular metabolism. To confirm the basic design of this method, an RSS-activated molecular donor was synthesized. As shown in Fig. 4b (top), the RSS reduces the nitro group to an amino group, which induces self-elimination to release H_2_Se. Fluorescence spectra indicated that the fluorescence signal of donors FITA-FD5 and FITA-FD6 can be detected with the addition of RSS. Furthermore, the fluorescence intensity achieved equilibrium within 30 min (Fig. 4b, bottom), demonstrating the validity and feasibility of this strategy.

In 2025, the Pluth's group developed a new class of H_2_Se donors based on isoselenocyanates (ISeC-R).^71^ As shown in Fig. 4c (top), different H_2_Se donors were synthesized from aniline derivatives. When the system was treated with Cys, the donor reacts with Cys to yield an intermediate, which subsequently undergoes intramolecular cyclization followed by β-elimination to liberate H_2_Se and a corresponding coproduct (Fig. 4c, bottom). To further evaluate the performance of the donor in H_2_Se generation, the cell imaging test was performed. With the fluorescence indicator, strong fluorescence was observed in cells treated with a combination of the donor and Cys (Fig. 4d), confirming that this donor can be effectively activated to release H_2_Se within a cellular environment.

Based on a similar mechanism, Zhu and co-workers designed and synthesized isoselenocyanate-based donor (IN-D) to visualize H_2_Se release.^72^ As shown in Fig. 4e, the donor undergoes a selective nucleophilic attack by the sulfhydryl group of Cys on the N=C=Se bond, triggering two parallel reaction pathways. In Pathway I, the enhancement of the intramolecular charge transfer (ICT) effect yields a strong fluorophore alongside the release of H_2_Se, enabling the monitoring of H_2_Se in biological environments. In Pathway II, the reaction produces an organic amine and 2-selenothiazolidine-4-carboxylic acid without the liberation of H_2_Se. Additionally, the study concurrently developed a near-infrared (NIR) fluorescent probe for sensing H_2_Se liberated from this donor. As shown in Fig. 4f, live cells incubated with IN-D and Cys exhibited enhanced fluorescence (red fluorescence in c2 and c3), and the signal intensity also increased with the concentration of IN-D. These results confirmed that this donor based on isoselenocyanate can release H_2_Se in a biological environment.

**Fig. 5:**
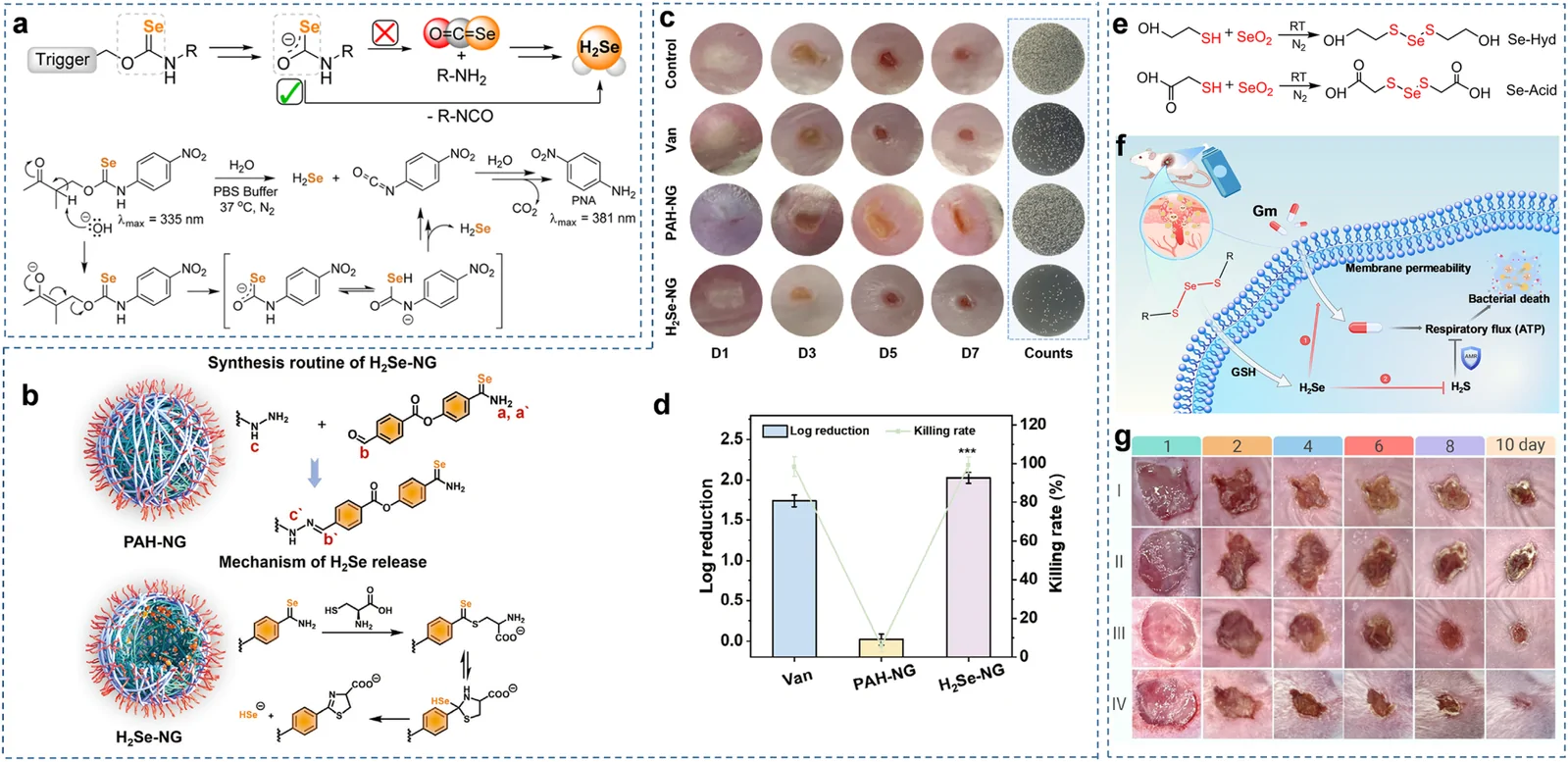
(a) Reaction mechanism of H_2_Se donor based on selenocarbamate (top), and the release mechanism of H_2_Se donor in buffer. Reproduced with permission from ref. 73. Copyright 2023, Royal Society of Chemistry. (b) Synthesis routine and release mechanism of nanogel-based H_2_Se donor. Reproduced with permission from ref. 74. Copyright 2024, Wiley-VCH. (c) Images of mice abscess sites after being treated with different therapeutic agents. Reproduced with permission from ref. 74. Copyright 2024, Wiley-VCH. (d) Bacterial counts and killing rate of the infected tissue obtained from different mice models. Reproduced with permission from ref. 74. Copyright 2024, Wiley-VCH. (e) Synthesis routine of H_2_Se donors based on S–Se–S bond. Reproduced with permission from ref. 75. Copyright 2024, Innovation Press. (f) Schematic illustration of the Se-Acid relieve H_2_S-induced bacterial resistance. Reproduced with permission from ref. 75. Copyright 2024, Innovation Press. (g) Images of mice abscess sites after being treated with different therapeutic agents (I: PBS; II: Gm; III: Se-Acid; IV: Gm + Se-Acid). Reproduced with permission from ref. 75. Copyright 2024, Innovation Press.

In 2023, Pluth's group reported a class of selenocarbamate-based H_2_Se donors,^73^ which release H_2_Se directly through a triggered self-immolative cascade reaction. As illustrated in Fig. 5a (top), once the trigger is removed by the external target, the resulting selenocarbamate undergoes intramolecular rearrangement to directly generate H_2_Se. To verify this hypothesis, a γ-ketothiocarbamate was selected as the response site. As shown in Fig. 5a (bottom), OH^−^ acts as a nucleophile to react with the γ-ketothiocarbamate and induces deprotonation at the β-position. The as-formed selenocarbamate then undergoes selective C–Se bond cleavage, leading to the direct release of H_2_Se and *p*-nitroaniline. The key feature of this mechanism is that the selenocarbamate will directly release H_2_Se and not form the intermediate C=O=Se.

Apart from molecular tools, there is also report of polymeric micelles integrated with H_2_Se donors for the treatment of multidrug-resistant (MDR) bacterial infections. As shown in Fig. 5b (top), Li's group developed a nanogel-based delivery system (H_2_Se-NG) by conjugating PAH-NG and SeAs using a hydrazone bond.^74^ In the presence of Cys, nucleophilic attack on the C=Se site of SeAs results in the loss of NH_3_, triggering the formation of a thiazolidine intermediate, which subsequently undergoes intramolecular nucleophilic reaction to release H_2_Se (Fig. 5b, bottom). To evaluate the therapeutic efficacy of this H_2_Se donor, a mouse model of subcutaneous abscess was established. The *in vivo* results indicated that the H_2_Se-NG and vancomycin groups exhibited recovered skin at the initial challenge sites, characterized by the complete absence of observable abscesses (Fig. 5c, the second and fourth line). In contrast, the control and PAH-NG (without H_2_Se-generation site) groups still exhibited significant crusting. By homogenizing the infected tissues, H_2_Se-NG exhibited a 98.4% bacterial killing rate (Fig. 5d), further demonstrating that H_2_Se-NG effectively inhibits bacterial proliferation by interfering with sulfur metabolism and antioxidant stress mechanisms.

Wang's group reported a novel H_2_Se donor based on an S–Se–S scaffold,^75^ designed to reverse antibiotic resistance in bacteria. Two core compounds, Se-Hyd and Se-Acid, were successfully synthesized *via* a one-pot method under a nitrogen atmosphere (Fig. 5e). In the presence of GSH, GSH will attack on the β-selenium atom of Se-Acid *via* nucleophilic reaction, yielding 2-mercaptoacetic acid (TGA) and the intermediate TGA-Se-SG. Subsequently, this intermediate reacts with GSH to produce TGA-I and oxidized glutathione (GSSG). Ultimately, TGA-I undergoes a cascade transformation with excess GSH to release the H_2_Se. The released H_2_Se exerts its effects through a dual-action pathway: first, it induces cell membrane depolarization and disrupts membrane integrity, thereby enhancing membrane permeability and promoting the intracellular accumulation of antibiotics such as gentamicin (Gm). Second, acting as an H_2_S mimetic (or H_2_S camouflage agent), it competitively binds to targets, reversing the inhibition of the respiratory electron transport chain typically caused by H_2_S. This restores bacterial respiratory flux and increases ATP levels, providing the necessary energy for the active transport of antibiotics (Fig. 5f). To evaluate the *in vivo* bactericidal efficacy of Se-Aid, it was applied to the treatment of skin wounds in mice. As shown in Fig. 5g, compared with the other control groups, the mice treated with Se-Aid exhibited superior wound healing (the fourth line), demonstrating advantages for the treatment of drug-resistant wound infections. This strategy effectively eliminates H_2_S-mediated resistance phenotypes and significantly enhances antibacterial efficacy.

Utilizing biothiols, esterases, or biomarkers as nucleophilic triggers enables the on-demand release of H_2_Se. Benefiting from modular structural programmability, distinct activation cascades, and excellent biocompatibility, these donor platforms enable spatiotemporally controllable H_2_Se release at the intracellular level and even within specific lesion sites, thereby serving as a powerful toolkit for interrogating the biological functions of H_2_Se. Nevertheless, these donor platforms are still constrained by several bottleneck issues. First, their heavy reliance on high-abundance endogenous thiols, such as Cys and GSH, inevitably compromises their fidelity in complex cellular redox networks. Second, because the release process frequently involves multi-step cascades or complex intermediate transformations, the actual H_2_Se release efficiency is difficult to quantify. Third, the concomitant generation of bioactive by-products often introduces additional variables, complicating the elucidation of the intrinsic physiological actions of H_2_Se. Furthermore, most designs still depend on a singular stimulus-responsive mechanism, which compromises their selectivity and tissue-targeting capabilities within heterogeneous disease microenvironments. In short, superior H_2_Se donors must evolve toward integrating higher selectivity, predictable kinetics, multiplexed stimuli-responsiveness, and quantitative tracking capacity within a unified platform.

### 3.3 Other reaction mechanisms

The aforementioned H_2_Se donors possess potent antioxidant activity across both *in vitro* and cellular models, encompassing the scavenging of ROS, the maintenance of redox homeostasis, and the upregulation of antioxidant proteins. However, the hydrolysis or nucleophilic reactions are highly susceptible to interference from various species in complex biological environments,^76^ which affects the H_2_Se generation rate. Thereby researchers have developed novel triggering strategies based on photoexcitation or free-radical chain reactions. By leveraging exogenous signals or specific chemodynamic processes, these mechanisms achieve precise H_2_Se release and deep-tissue imaging, significantly expanding the application of these donors.

**Fig. 6:**
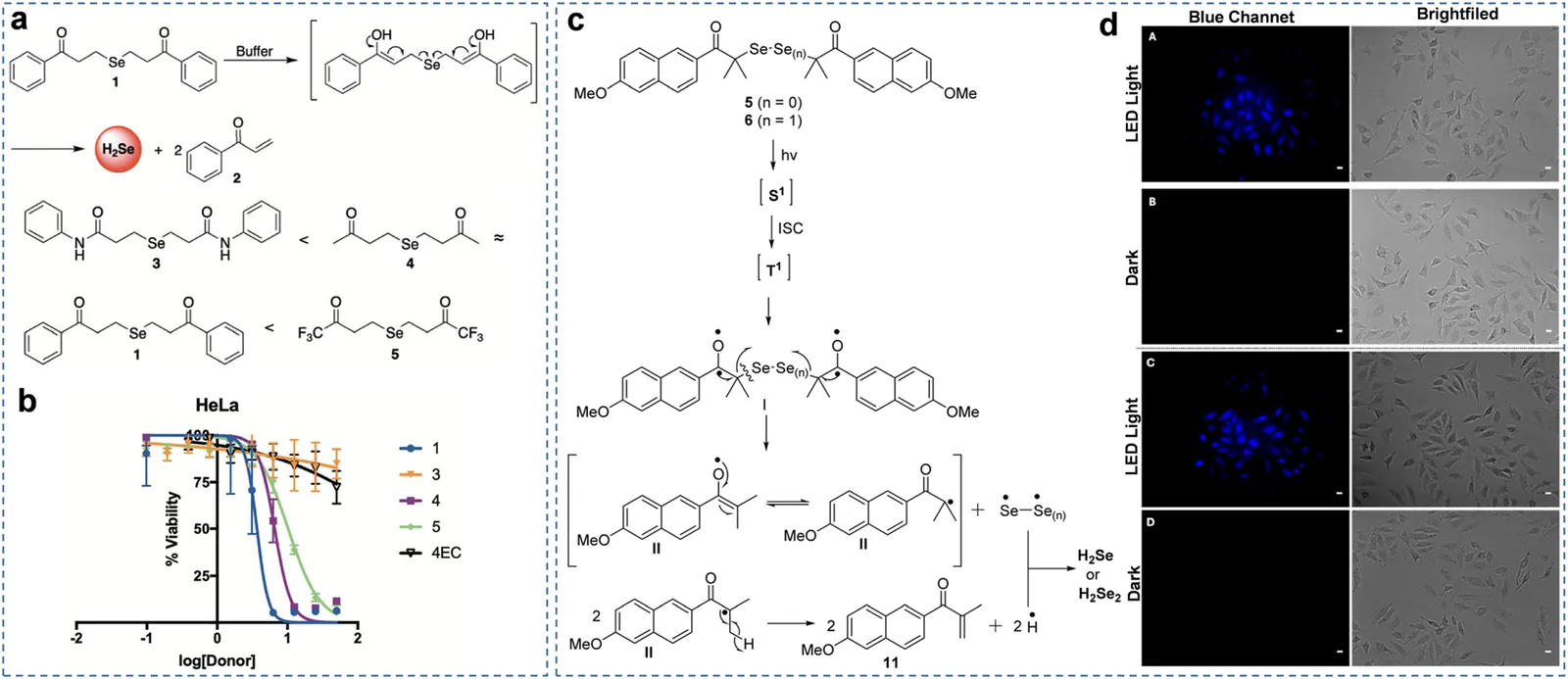
(a) Reaction mechanism of H_2_Se donor based on γ-ketoselenides (top), and different kinds of H_2_Se donors with increasing alpha proton acidity. Reproduced with permission from ref. 77. Copyright 2022, Royal Society of Chemistry. (b) Cell growth inhibition of H_2_Se donors in HeLa cells. Reproduced with permission from ref. 77. Copyright 2022, Royal Society of Chemistry. (c) The mechanism of H_2_Se_2_/H_2_Se formation from photoactivated H_2_Se donors. Reproduced with permission from ref. 78. Copyright 2025, Royal Society of Chemistry. (d) Fluorescence images of cells treated with compounds 5 (the first and second line) and 6 (the third and fourth line) with and without photoexcitation. Reproduced with permission from ref. 78. Copyright 2025, Royal Society of Chemistry.

In 2022, Lukesh's group designed and synthesized a class of pH-responsive γ-ketoselenides,^77^ which are capable of the site-specific release of H_2_Se. As shown in Fig. 6a (top), under neutral to weakly alkaline conditions, the γ-ketoselenide initially undergoes α-carbon deprotonation to form an enolate intermediate, which triggers a β-elimination reaction. This process leads to the dissociative release of H_2_Se and the simultaneous generation of a corresponding enone by-product. Experimental results demonstrated that an alkaline environment significantly accelerates the release of H_2_Se by promoting the α-deprotonation, whereas weakly acidic conditions effectively inhibit this process (Fig. 6a, bottom). Live cells were incubated with different kinds of H_2_Se donors to investigate the growth inhibition activity. As shown in Fig. 6b, donors 1, 4, and 5 exhibited significant inhibitory effect on cell proliferation, suggesting that these H_2_Se donors can effectively release H_2_Se in live cells.

Although photo-activated H_2_Se donors have been previously reported by the Pluth and Yi groups, their release mechanisms typically do not involve the direct photo-induced cleavage of the C–Se bond. Drawing inspiration from the efficient photo-induced C–S bond cleavage of naphthoyl-based sulfur compounds, Xian's group synthesized a series of monoselenium (5) and diselenium (6) compounds as photo-activated H_2_Se donors (Fig. 6c).^78^ Upon photoexcitation, compounds 5 and 6 undergo an n → π* transition to reach the singlet excited state, followed by efficient intersystem crossing (ISC) to the triplet state. In this high-energy state, the carbonyl group transforms into a biradical intermediate (I), which induces the homolytic cleavage of the C–Se bond, generating intermediate II and a selenium radical. Intermediate II further releases a hydrogen radical to form the stable product 11, while the hydrogen and selenium radicals couple to ultimately yield H_2_Se or hydrogen diselenide (H_2_Se_2_). Cellular imaging experiments were performed to validate the biological application potential of this system. As shown in Fig. 6d, live cells treated with photo-irradiation exhibited significant blue fluorescence, whereas the untreated group exhibited no detectable signal. These results confirm that compounds 5 and 6 enable spatiotemporally controllable release of H_2_Se intracellularly, effectively overcoming the limitations of traditional donors, such as sluggish release kinetics, interference from complex biological systems, and the lack of key intermediates (*e.g.*, C=O=Se).

**Fig. 7:**
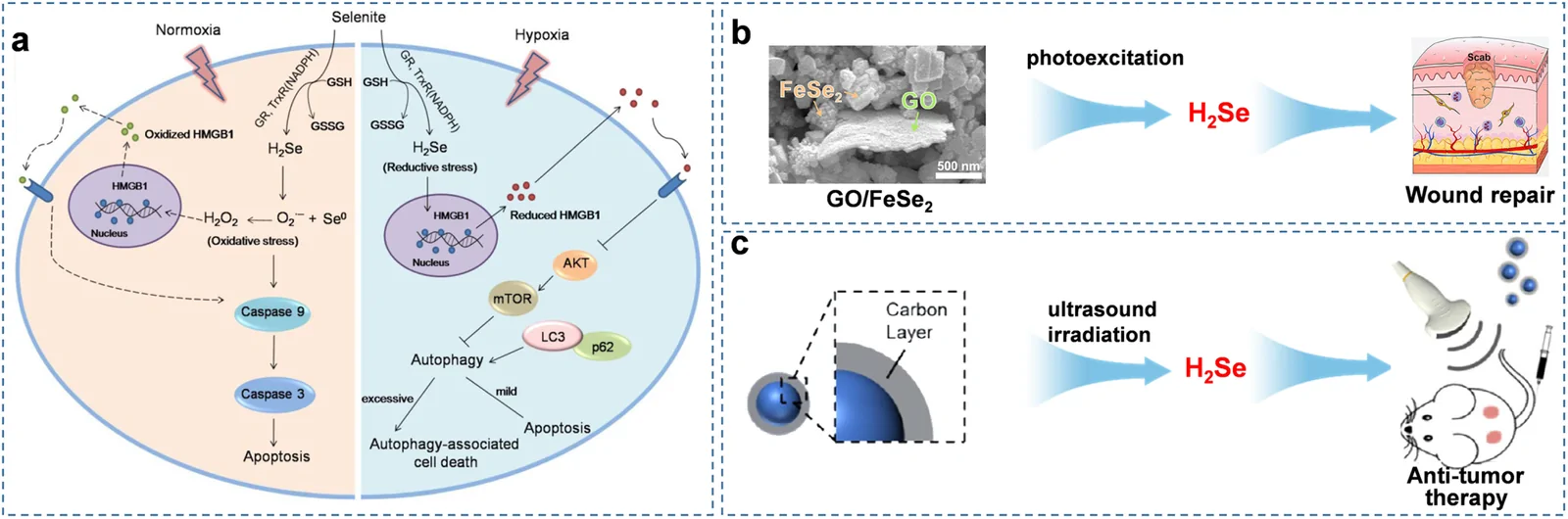
(a) Proposed divergent mechanisms of selenite metabolism and signalling under normoxia and hypoxia. Adapted with permission from ref. 79. Copyright 2019, Ivyspring International Publisher. (b) A photoactivated GO/FeSe_2_ nanoplatform for H_2_Se generation and wound healing. Adapted with permission from ref. 80. Copyright 2024, Elsevier. (c) An ultrasound-responsive Cu_2−*x*_Se@cMOF nanoplatform for H_2_Se generation and cancer therapy. Adapted with permission from ref. 81. Copyright 2024, American Chemical Society.

Inorganic salts and nanomaterials are also extensively utilized in the fabrication of H_2_Se-releasing systems. For instance, Tang's group reported that sodium selenite (Na_2_SeO_3_) exhibits distinct biological effects on HepG_2_ cells under normoxic (20% O_2_) and hypoxic (1% O_2_) conditions.^79^ As shown in Fig. 7a, Na_2_SeO_3_ can enter in the cells and be converted into H_2_Se through a metabolic pathway under normoxic condition, then the H_2_Se will react with superoxide anions (O_2_^−^) to produce hydrogen peroxide (H_2_O_2_), thereby inducing oxidative stress. During this process, the autophagy associated protein was activated to trigger apoptosis. In contrast, under hypoxic conditions, the intracellular accumulation of H_2_Se induces reductive stress and can reduce proteins to their sulfhydryl (reduced) forms. Furthermore, H_2_Se triggers autophagy by inhibiting the Akt/mTOR signalling pathway. This study establishes the role of Na_2_SeO_3_ as an exogenous H_2_Se donor and elucidates the regulatory mechanism of active small molecules.

Deng's group developed a H_2_Se-evolving nanoparticle composed of graphene oxide (GO) and FeSe_2_ (Fig. 7b).^80^ Triggered by acidity and light irradiation, this nanodonor facilitates the release of H_2_Se. Notably, it exhibits excellent wound repair capability *in vivo*. Jiang's group developed an integrated therapeutic nanomaterial (Fig. 7c),^81^ Cu_2−*x*_Se@cMOF, which generates H_2_Se under ultrasound irradiation. By simultaneously producing copper ions and ROS, this nanoplatform integrates sonodynamic therapy (SDT), cuproptosis, and gas therapy to achieve effective tumor treatment *in vivo*.

These H_2_Se donors represent a significant platform beyond classical hydrolysis and nucleophile-triggered systems, offering enhanced spatiotemporal precision, multi-modal triggering, and integrated therapeutic functionality. For instance, inorganic selenite donors have extended the utility of H_2_Se delivery into synergistic cancer therapies validated *in vivo*. However, several challenges remain to be addressed. The absence of standardized quantitative release kinetics across different donor classes hinders systematic comparison of their biological efficacy. Also, similar to other donors, the potential cytotoxicity of by-products generated from H_2_Se release has not been evaluated. Therefore, the development of donor systems characterized by finely tailored release kinetics, augmented tissue penetration depth *via* near-infrared photo-activation, and rigorous *in vivo* metabolic safety assessments will be of great significance in the future.

## 4. Synthesis and biological application of H_2_Se fluorescent probes

Similar to H_2_S, H_2_Se acts as a key endogenous mediator that modulates various physiological functions, particularly in maintaining cellular redox homeostasis.^82,83^ However, there are distinct differences between these two species. Compared with H_2_S, H_2_Se possess enhanced nucleophilicity and reducing capacity due to the higher polarizability of Se,^30,31^ which make it easy to degrade and react with other biomacromolecules, such as proteins.^84–86^ Moreover, H_2_Se exhibits Janus properties in biological system at different concentrations. Trace amounts of H_2_Se will interact with specific proteins to participate in intracellular signalling, thereby maintaining normal physiological activities.^87^ However, when H_2_Se levels exceed the physiological normal standard, the excess H_2_Se perturbs cellular homeostasis, leading to functional impairment and eventually programmed cell death.^88^ Thus, real-time monitoring of the fluctuations of H_2_Se in biological system is important for accurately evaluating the physiological functions.

Various traditional detection methods, such as hydride generation-atomic fluorescence spectrometry (HG-AFS),^89,90^ nitrogen microwave inductively coupled atmospheric-pressure plasma (MICAP),^91,92^ colorimetric methods and neutron activation analysis (NAA),^93–97^ have been extensively used for the detection of biologically relevant selenides. While these techniques rely on elemental conversion (atomization, ionization, or nuclear reaction) for quantification, they inherently preclude the non-invasive, non-destructive evaluation of molecular H_2_Se in living systems. Significantly, fluorescence methods exhibit high sensitivity, superior spatiotemporal resolution, and excellent biocompatibility,^98–103^ and as such provide a more sophisticated means for the real-time tracking of biogenic selenides within living organisms.

To date, several H_2_Se-responsive fluorescent probes based on different recognition sites have been developed to sense H_2_Se in live cells and *in vivo*. In the following sections, the rational design and synthesis of H_2_Se-responsive fluorescent probes will be described, and their performance for the fluorescence imaging of H_2_Se in complex biological scenarios will be outlined.

### 4.1 Benzoselenadiazole-based fluorescent probes

**Fig. 8:**
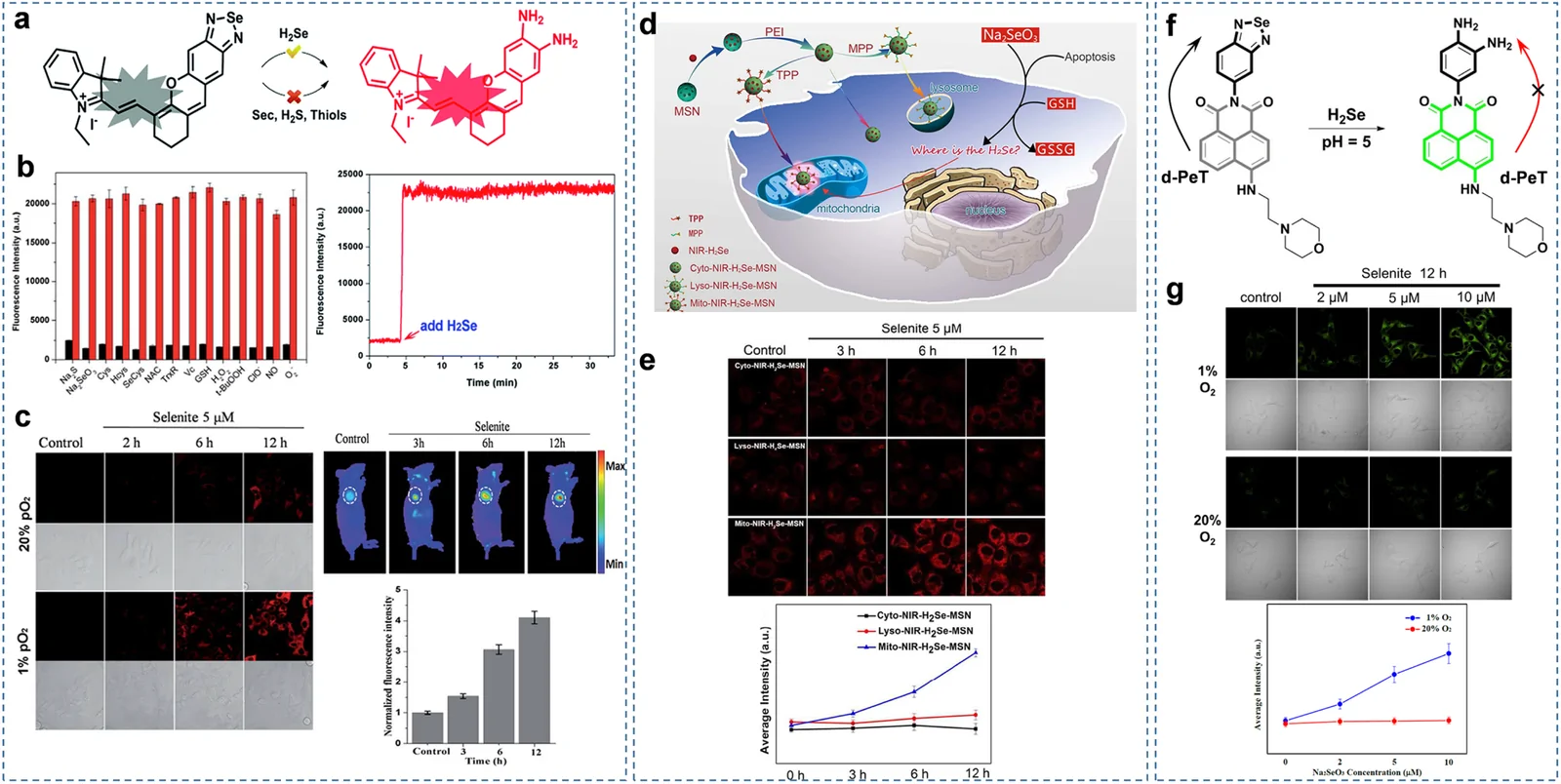
(a) The response mechanism of benzoselenadiazole-based fluorescent probe toward H_2_Se. Reproduced with permission from ref. 104. Copyright 2016, Royal Society of Chemistry. (b) The selectivity and reaction kinetic of this probe. Reproduced with permission from ref. 104. Copyright 2016, Royal Society of Chemistry. (c) Fluorescence imaging of H_2_Se in live cells and *in vivo* when the samples were treated with selenite. Reproduced with permission from ref. 104. Copyright 2016, Royal Society of Chemistry. (d) Schematic illustration of the subcellular targeting fluorescent probes imaging organellar H_2_Se. Reproduced with permission from ref. 105. Copyright 2018, American Chemical Society. (e) Fluorescence imaging of subcellular H_2_Se in live cells upon treatment of selenite. Reproduced with permission from ref. 105. Copyright 2018, American Chemical Society. (f) The response mechanism of lysosome-targeted fluorescent probe toward H_2_Se. Adapted with permission from ref. 107. Copyright 2013, Royal Society of Chemistry. (g) Fluorescence imaging of lysosomal H_2_Se in live cells upon treatment of selenite under normoxic and hypoxic conditions. Reproduced with permission from ref. 107. Copyright 2013, Royal Society of Chemistry.

In 2016, Tang's group discovered that selenol can react with 2,1,3-benzoselenadiazole to cleave the Se–N bond *via* nucleophilic addition,^104^ and that the 2,1,3-benzoselenadiazole was an effective fluorescence quenching group due to the heavy-atom effect of Se. Thus, the first H_2_Se-responsive fluorescent probe (NIR-H_2_Se) was developed with benzoselenadiazole as trigger for sensing H_2_Se in live cells and *in vivo*. As shown in Fig. 8a, the merocyanine dye was modified with 2,1,3-benzoselenadiazole and the fluorescence is quenched. The probe can selectively react with H_2_Se over Sec, H_2_S and other small molecule thiols, and the fluorescence can be activated by formation of the bis aromatic amine. Also, this fluorescent probe exhibited excellent selectivity to H_2_Se but not ROS and other biothiols (Cys, Hcys and GSH), and the fluorescence reached a plateau within seconds, which is crucial for the real-time tracking of transient H_2_Se fluctuations (Fig. 8b). The applicability of this probe was assessed in live cells and *in vivo*. From Fig. 8c, after being treated with sodium selenite, the fluorescence intensity increased with increasing incubation time. Moreover, live cells under normal oxygen content (20%) exhibited much weaker fluorescence compared with cells in a hypoxic environment, demonstrating that the content of H_2_Se scavenges surplus endogenous ROS acting as a redox mediator. Endogenous H_2_Se in tumours has been sensitively imaged without background interference (Fig. 8c), further indicating that this probe can be used to image H_2_Se in biological systems. As the pioneering study in H_2_Se fluorescence sensing, this work marks a significant milestone, paving the way for real-time exploration of the biological roles in complex living systems.

To further confirm the distribution of H_2_Se in subcellular organelle, Tang's group constructed various subcellular targeting fluorescent probes by loading the H_2_Se-responsive small molecular probe (NIR-H_2_Se) in mesoporous silica nanoparticles (Fig. 8d).^105^ From the cell imaging results, when live cells pretreated with sodium selenite and then incubated with three kinds of fluorescent probes (cytoplasm, lysosome and mitochondria-targeting probes), the mitochondria exhibited enhanced fluorescence with increased incubation time and the signal was more intense than the cytoplasm and lysosome (Fig. 8e), demonstrating that H_2_Se was generated in the mitochondria of live cells under the stimulation of exogenous interferences. This work delivers a powerful molecular tool to reveal the distribution of H_2_Se, offering a more profound understanding of H_2_Se physiological functions, particularly its regulatory roles in redox signalling networks.

Subsequently, Tang's group developed a ratiometric fluorescent probe system to synchronously detect H_2_Se and O_2_^·–^ in tumour cell.^106^ The results demonstrated that the concentration of O_2_˙^−^ maintained low levels during treatment with Na_2_SeO_3_ under hypoxic conditions, but a marked increase in the intracellular H_2_Se content was observed, which illustrated that Na_2_SeO_3_ can induce reductive stress to perturb the cellular redox homeostasis for anti-tumour treatment. Based on the response site of benzoselenadiazole, Zhang's group reported a lysosome-targeted fluorescent probe to image the change of lysosomal H_2_Se under hypoxic conditions.^107^ As shown in Fig. 8f, the fluorescence of the probe can be effectively quenched due to intramolecular photoinduced electron transfer (PeT), but when the Se–N bond was broken by H_2_Se to release the Se atom, the PeT effect was inhibited to enhance the fluorescence. Specially, this fluorescent probe can aggregate in the lysosome. Under a hypoxic microenvironment, the fluorescence intensity gradually increased when the concentration of the sodium selenite increased, and the signal maintained steady under normal oxygen concentrations (Fig. 8g), further indicating that the lysosomal H_2_Se levels are intimately correlated with intracellular oxygen content.

Although benzoselenadiazole-based probes have been used for H_2_Se imaging, several reports have also leveraged this specific scaffold to construct GSH- and DTT-responsive systems. Consequently, the signalling fidelity of such probes within the thiol-rich intracellular environment need to be further investigated.

### 4.2 1,2-Dithiane ring-based fluorescent probe

**Fig. 9:**
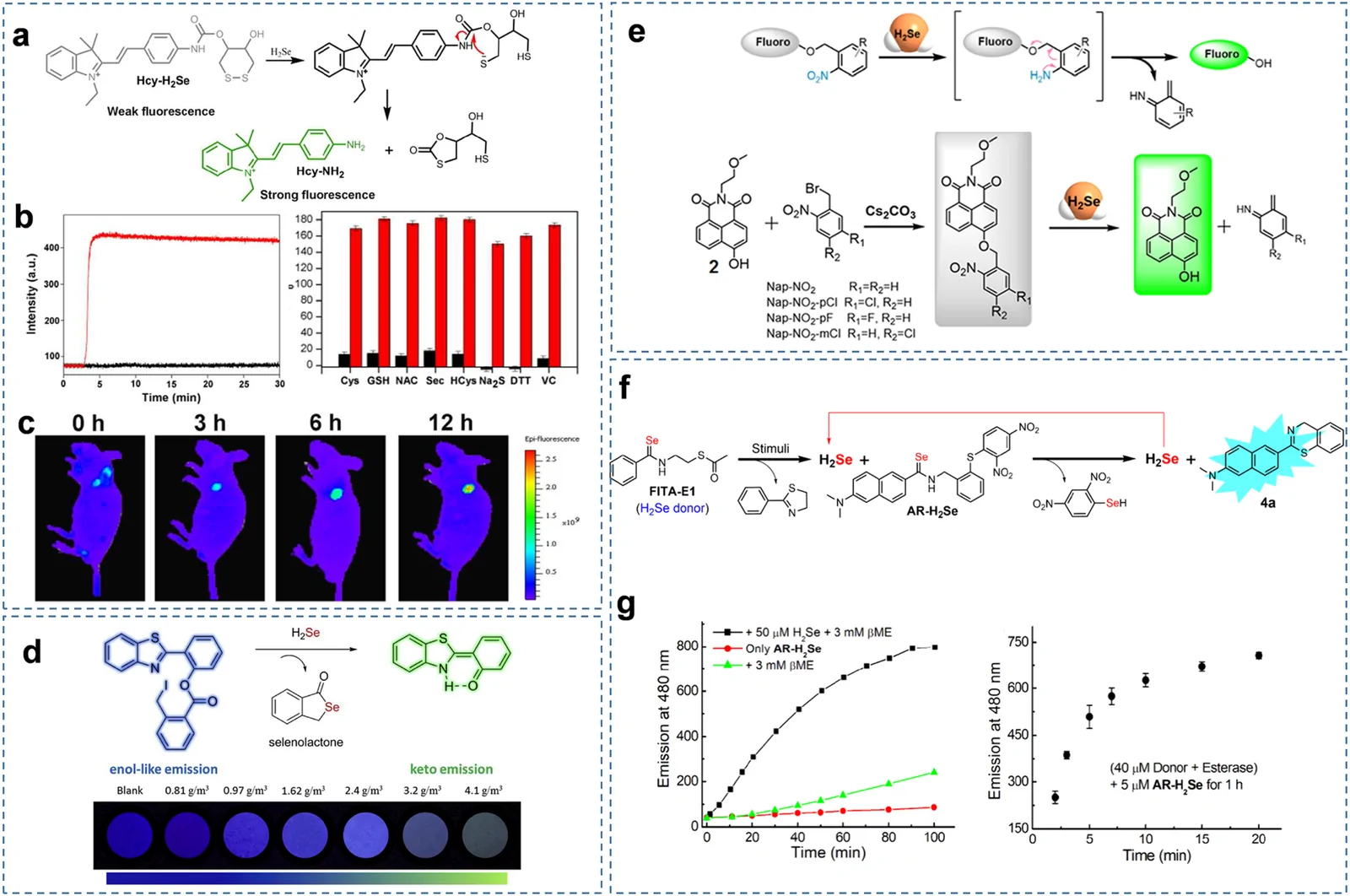
(a) The response mechanism of 1,2-dithiane ring-based fluorescent probe toward H_2_Se. Reproduced with permission from ref. 110. Copyright 2017, American Chemical Society. (b) The reaction kinetic and selectivity of this probe. Reproduced with permission from ref. 110. Copyright 2017, American Chemical Society. (c) Fluorescence imaging of H_2_Se *in vivo* at different time point. Reproduced with permission from ref. 110. Copyright 2017, American Chemical Society. (d) The response mechanism of 2-(iodomethyl)benzoate-based fluorescent probe toward H_2_Se, and the environmental H_2_Se was detected with portable paper-based sensor. Reproduced with permission from ref. 111. Copyright 2020, Elsevier. (e) The response mechanism of nitrobenzene-based fluorescent probe toward H_2_Se. Reproduced with permission from ref. 112. Copyright 2025, Royal Society of Chemistry. (f) The response mechanism of analyte-replacement fluorescent probe toward H_2_Se. Reproduced with permission from ref. 70. Copyright 2025, American Chemical Society. (g) Reaction kinetics of analyte-replacement fluorescent probe under different conditions. Reproduced with permission from ref. 70. Copyright 2025, American Chemical Society.

Despite the specificity of the reported benzoselenadiazole, the structural disassembly of the probe upon responding to H_2_Se unavoidably liberates native Se atoms, which can exert untoward perturbations on homeostatic cellular metabolism.^108,109^ Motivated by these concerns, Tang's group reported a second generation of H_2_Se-responsive fluorescent probes using a 1,2-dithiane ring as the new recognition unit.^110^ As shown in Fig. 9a, a hemicyanine dye was used as the signal unit and scaffold to link with a 1,2-dithiane ring to prepare the fluorescent probe (Hcy-H_2_Se). Then H_2_Se can react with the 1,2-dithiane to form sulfydryl, which will then attack as a nucleophile the intracellular carbonyl group, triggering an intramolecular cyclization that subsequently leads to the cleavage of the amide linkage and liberation of the hemicyanine. The probe achieves maximum H_2_Se response within seconds without being triggered by other biothiols (Fig. 9b), validating its rapid reaction kinetics and high selectivity. The *in vivo* fluorescence images indicated that Hcy-H_2_Se can image H_2_Se generated from sodium selenite in hypoxic solid tumours (Fig. 9c). This work not only provide a novel fluorescent probe to image H_2_Se, but also offers a robust strategy that can be extended to the development of other chalcogen-specific sensing platforms. Admittedly, in such tandem multi-step activatable systems, any intermediate accumulation or competitive side-reactions will distort the signal. Furthermore, the strong nucleophilicity of open-chain dithiol intermediates renders it susceptible to rapid reactions with intracellular electrophiles, thereby lowering cyclization efficiency and signalling sensitivity.

### 4.3 2-(Iodomethyl)benzoate-based fluorescent probes

The Se–N and cyclic S–S bond can be specifically cleaved by H_2_Se, and various kinds of fluorescent probes towards H_2_Se have been developed. However, the high concentration of sulfhydryl-containing compounds will consume the fluorescent probe when used in biological systems, leading the limited sensitivity. Thus, in 2020, Zhang's group developed a 2-(2-hydroxyphenyl)benzothiazole-based fluorescent probe (HBT-Se).^111^ The hydroxyl of the fluorophore was modified with a recognition group and the exited state intramolecular proton transfer (ESIPT) process was blocked, accompanied by blue fluorescence. In the presence of H_2_Se, the 2-(2-hydroxyphenyl)benzothiazole was liberated to trigger the tautomerism of the intrinsic keto–enol, thus long wavelength fluorescence (green fluorescence) was observed (Fig. 9d). In this work, the author expanded the application of HBT-Se to environmental analysis. By doping the HBT-Se into a polyvinylidene fluoride microporous filter membrane, this portable paper-based sensor enables the sensitive detection of toxic H_2_Se gas, serving as a cost-effective and efficient sensing device for real-time monitoring air quality. Crucially, the ESIPT pathway dictates the formation of an intramolecular hydrogen bond, which undergoes pronounced inhibition in polar solvents. This intrinsic limitation inherently restricts the probe's performance for aqueous H_2_Se sensing, rendering such systems more suited for deployment in portable sensing devices rather than intricate biological systems.

### 4.4 Nitrobenzene-based fluorescent probes

Pluth and co-workers discovered that nitroarenes could be reduced into corresponding amino derivatives in the organic phase under Se catalysis.^73^ Based on this, Sun's group developed a novel strategy to construct a series of H_2_Se-responsive fluorescent probes based on nitroaromatic reduction.^112^ As shown in Fig. 9e, the fluorophore was covalently linked with nitroarenes through a self-immolative linker and the fluorescence was quenched because of the electron-withdrawing effect. An amino group was formed after reaction with H_2_Se, which undergoes a 1,4-elimination reaction to release the fluorophore. Different substituted nitroaromatics were investigated to confirm the reaction kinetics. The results indicated that a chlorine substituent at the *para*-position to the nitro group will create an effective H_2_Se-responsive fluorescence, which can respond to H_2_Se due to the electron-withdrawing properties of the chlorine. The incorporation of a nitrobenzene moiety as a specific H_2_Se recognition site further expands the repertoire of design strategies for developing high-performance fluorescent probes. However, it is noteworthy that within the tumour hypoxic microenvironment, the nitrobenzene moiety can also be reduced by overexpressed nitroreductase (NTR), thereby eliciting false positive signals.

### 4.5 Analyte-replacement fluorescent probes

An ideal sensing platform should facilitate the visualization of low-abundance targets without depleting the target pool, thereby ensuring minimal perturbation to the physiological redox equilibrium.^113^ Pluth's group reported an analyte-replacement sensing platform that responds to H_2_S accompanied by the donation of H_2_S, thus trace amount of H_2_S can be detected by signal amplification.^114^ Motivated by this work, Yi's group developed an arylthiol-activated arylselenoamide-based platform for the simultaneous detection and release H_2_Se.^70^ As illustrated in Fig. 9f, upon liberation from the donor, the H_2_Se reacts with the nitrobenzene moiety of the fluorescent probe (AR-H_2_Se), triggering the release of H_2_Se (scavenging/signalling) and the simultaneous formation of a thiazine-containing fluorophore. From the results (Fig. 9g), AR-H_2_Se remains in a fluorescence-quenched state with minimal background signal in the absence of H_2_Se. However, upon the addition of the H_2_Se, a progressive enhancement in fluorescence intensity was observed (left). This probe can be used to monitor the H_2_Se released from donor (right), demonstrating that the AR-H_2_Se was an effective fluorescence tool to sense the low-abundance of H_2_Se. By the same token, in platforms utilizing multi-step sequential activation for H_2_Se sensing and delivery, the intracellular stability and lifetime of intermediates (*e.g.*, aryl amino sulfides and free aryl thiols) remain unknown, thus their precise impact on the overall activation cascade efficiency has yet to be elucidated.

## 5. Conclusions and outlook

### 5.1 Conclusions

In this review, we have summarized research on H_2_Se chemical donors and fluorescent probes, providing a detailed overview of the synthetic strategies and release mechanisms of H_2_Se molecular donors. Additionally, the applications of H_2_Se fluorescent probes for cellular and *in vivo* imaging were also discussed. A diverse array of sources, including organic molecular donors based on nucleophilic or hydrolysis reactions, along with water-soluble selenites and nanoparticles, can all be utilized for H_2_Se generation in both solution and biological systems. In particular, the selective release of H_2_Se under specific stimuli further expands the application of these donors. Fluorescent probes based on benzoselenadiazole, 1,2-dithiane ring, 2-(iodomethyl)benzoate, and nitrobenzene moieties have been synthesized for the quantitative analysis of H_2_Se. Leveraging the high spatiotemporal resolution of fluorescence imaging, these imaging tools facilitate the real-time visualization of H_2_Se fluctuations in both cellular and *in vivo* environments. Advances in H_2_Se donors and fluorescent probes have elucidated its multifaceted functions, including cytoprotective effects, antioxidant properties, and signalling pathways. These developments offer a solid theoretical foundation for unravelling the underlying mechanisms of H_2_Se in pathological processes and therapeutic interventions.

### 5.2 Outlook

Despite significant progress having been made in the field of H_2_Se donors and fluorescent probes, several challenges remain to be addressed in future research to meet the demands of practical applications.

(1) Stimuli-responsive donors achieve the precise and controlled delivery of H_2_Se in living organisms upon activation by specific targets. However, the generated H_2_Se is susceptible to consumption through reactions with other biomolecules because of the high reactivity. Achieving more precise, controlled release at subcellular regions will prevent the non-specific leakage of molecular donors in normal tissue, thereby significantly enhancing the therapeutic advantages of H_2_Se in disease treatment.

(2) Organic H_2_Se donors are designed to liberate H_2_Se upon triggering by specific stimuli (*e.g.*, pH, enzymes, or reactive molecules), but most of these processes are confined to solution systems. The complexity of biological environments may impair reaction efficiency, resulting in limited H_2_Se yields that are significantly lower than the theoretical values. Consequently, it remains challenging to achieve persistent H_2_Se delivery required for demanding biomedical tasks. Therefore, the development of more stable and efficient H_2_Se generating reactions is critical to the practical application of chemical donors.

(3) Although various fluorescent probes based on different recognition sites have been developed for H_2_Se sensing, the model of H_2_Se target signalling occurs in an equivalent reaction ratio (1 : 1) which inevitably limits the sensitivity. This limitation makes them difficult to achieve the highly sensitive detection of trace H_2_Se during specific physiological processes, hindering the real-time monitoring and quantitative analysis in complex biological systems. Developing signal-amplified probes with a 1:m signal output kinetics is a pivotal future goal to facilitate highly sensitive H_2_Se profiling in diverse pathological contexts.

(4) While the generation pathways of H_2_Se *in vivo* have been extensively studied, its subcellular localization remains poorly defined. Currently, H_2_Se fluorescent probes suffer from poor subcellular resolution, making it challenging to precisely capture detailed information regarding H_2_Se in specific cellular metabolic pathways. Additionally, the relatively short emission wavelengths of fluorescent probes preclude the effective visualization of H_2_Se dynamics in deep-tissue environments *in vivo.* Therefore, it is essential to develop superior fluorescent probes with long-wavelength emission and organelle targeting capabilities.

(5) Despite successful H_2_Se imaging in biological systems, the clinical relevance of H_2_Se in various diseases remains largely unexplored. As a vital small molecule involved in multiple physiological activities, the fluctuation of H_2_Se is intimately associated with the onset of diseases. Nevertheless, comprehensive studies in this area are currently limited. Therefore, further efforts are required to fully elucidate the underlying physiological mechanisms of H_2_Se, which remains a critical focus for future research.

In summary, the future landscape of H_2_Se research remains centred on its physiological delivery, therapeutic potential, and sophisticated bioanalytical sensing. As a key reactive signalling species, unravelling its complexities will offer new way for overcoming perennial hurdles in human disease management. We also believe that an increasing number of researchers will devote their efforts to this burgeoning field, catalysing more profound and extensive investigations of H_2_Se and unlocking its full potential in life sciences and medicine.

## Author contributions

Yibo Zhou contributed to the writing and editing of the manuscript. Pin Shang and Jun Zhang reviewed the manuscript. Zhihe Qing and Tony D. James guided, reviewed and edited the writing of this paper.

## Conflicts of interest

There are no conflicts to declare.

## Data Availability

All data described in this review are sourced from previously published literature, and no new data were generated as part of this work.
